# Authors' reply

**Published:** 2010

**Authors:** Jitendra K S Parihar, Devendra P Vats, Rakesh Maggon, Vijay Mathur, Anirudh Singh, Sanjay K Mishra

**Affiliations:** Department of Ophthalmology, Army Hospital (Research and Referral), Delhi Cantt -10, India

Dear Editor,

We thank and highly appreciate Murali for his interest in our article[1] and their insightful and well-articulated comments.

**Figure 1:**
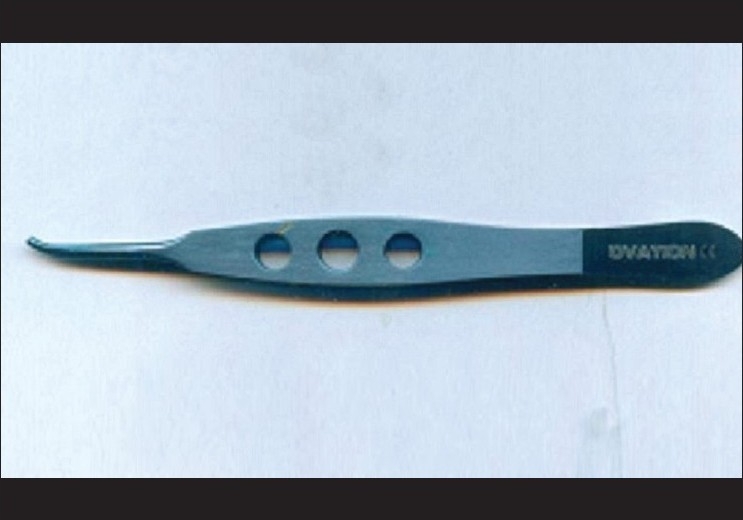
Modified AGV tube insertion forceps

**Figure 2:**
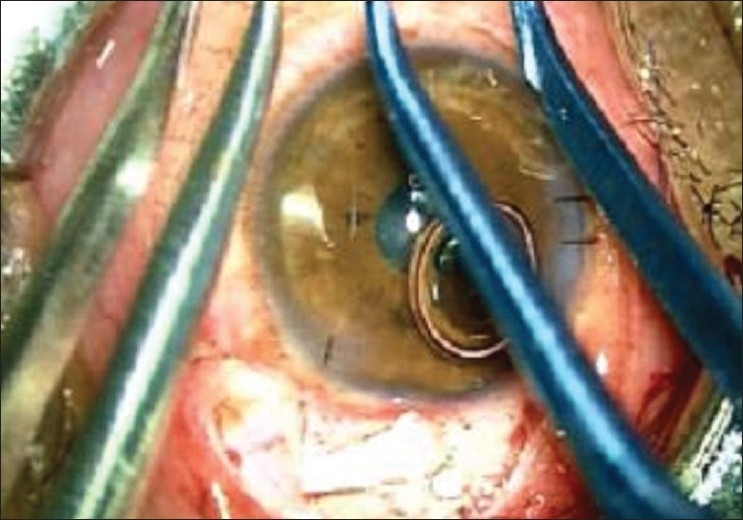
Modified AGV tube insertion forceps compared with Dr Ahmed's original forceps

**Figure 3:**
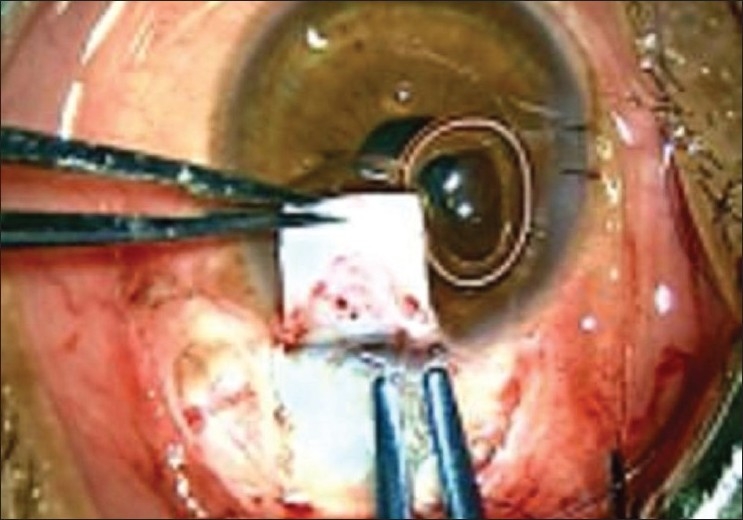
Insertion of AGV tube with the help of modified tube insertion forceps

Their observation regarding the insertion forceps is absolutely correct. In fact insertion forceps facilitate insertion of tube through 22/23 gauze needle track with precision and ease. Although we have not commented on it specifically, the insertion forceps have been used in all cases. In fact one of the photograph [Fig.6] in the original article, shows this instrument in use.[2] In our practice we found the insertion forceps designed by Dr Ahmed to be a little cumbersome while in use under moderate to high magnification of operating microscope. The author feels that these forceps are more heavy and large in size as compared to other microsurgical instruments being used in this surgery. Considering this observation, we have modified the insertion forceps which is manufactured by OVATION International, Jaipur (Rajasthan) India [Fig. 1]. This modified tube insertor forceps is made up of titanium, weighing 8g as compared to 22 g of the original forceps. The groove of the forceps is further modified to hold and insert the tube much more conveniently. Over and above, the forceps are much smaller in size, having a length of 110 mm as compared to 120 mm, while also retaining all the privileges of the original insertion forceps designed by Dr Ahmed [Figs. 2 and 3].

The tube extender is well reported in the literature[2] and has been used in situations as quoted by the authors. We have not had an occasion to use the extender as yet as none of our patients has had tube retraction to the extent that tube extension would be required.

The use of preserved allograft sclera is also well known, however, we are not inclined to use it routinely, since our experience has been that the use of these grafts results in increased postoperative inflammation. Over and above, the preserved allograft sclera invariably gives a discolored and elevated appearance under the cover of the conjunctiva. We have recommended constructing a scleral flap exactly in the manner that a scleral flap is constructed for trabeculectomy, which allows insertion of valve tube exactly through the site of the trabecular meshwork rather than anterior and proximal to the corneal endothelium or in proximity to the iris surface. In our view, tube insertion through site of trabecular meshwork is most ideal situation of tube into anterior chamber and minimizes the risk of postoperative complications and undue inflammation.
